# Report to the State Society of Michigan on the Diseases of Children

**Published:** 1858-06

**Authors:** A. B. Palmer


### *Report to the State Society of Michigan on the Diseases of Children.*

BY A. B. PALMER, M. D.

AT the last meeting of this Association I had the honor of being appointed to present a Report on the *Diseases of Children.* In discharging the duty imposed by that appointment, in the absence of any intimation as to the particular department of the general subject which it may have been thought desirable to have presented, and considering the fullness with which particular forms of Children's Diseases are treated of in the books accessible to all, I propose to present to your attention the importance of carefully studying these diseases, with a view, if possible, to diminish the extensive suffering and terrible mortality prevailing almost everywhere, among these little innocents of "tender age". Leaving then, for other hands or for other occasions, if the subject should be deemed worthy of being pursued, a special consideration of the peculiarities of the Diseases of Children, or the particular causes which operate in their production, the subject of this Report will be, *The great importance and urgent duty of specially studying the Diseases of Children.*

Our Profession is a benevolent one. To do good is our business. We are placed as guardians over the public health. We are to repel the enemies of human life. It is our special province to shield from danger and relieve the suffering of those who, from a want of knowledge or of power, are unable to protect or relieve themselves. The great danger, the utter helplessness, the total dependence, and the severe suffering of sick infancy, appeal most eloquently to our benevolence. We have all been children—dependent helpless children. We have all had a mother's care; and in our intercourse with the world, we have observed something of the all-pervading intensity of a mother's love. Many are fathers; and all have witnessed the calm, deep earnestness of a father's affection. As sympathizing integers of a common humanity—as men, as well as members of a benevolent Profession,—the sufferings and dangers of children are urged upon our attention.

But I propose to present the subject in a scientific and more strictly professional point of view.

The extensive prevalence, the great severity and fatal character of the Diseases of Children, render them subjects of intense professional interest.

As the result of careful investigation, and of accurate statistical record, it is found that throughout England and Wales, *one child in five dies within a year after birth, and one in three before the completion of the fifth year*. Taking into the account only larger towns and the metropolis of Great Britain, a much larger and more frightful proportion of infantile deaths, compared with the survivals, will be found.—According to these statistics, each young family containing three little children must expect to deposit one of them in a diminutive grave, and experience all the sadness of desolation—all the anguish of wounded affection—which such an event involves.

As to the infant mortality among us, in the absence of proper Registration Laws, we are unable to speak with the same particularity and precision. Some of the older States, it is true, have Registration Laws in force, but none of them have the perfection of those of England and Wales; and in our own State (I say it in sorrow and in shame), the subject, except as served by individuals, is totally neglected. Even in this city, I have sought, through a medical friend, in vain, for records which would indicate the number of children that have died during the past year, as compared with the whole mortality! Whether it is made the duty of any officers to keep such records, I have not learned, but, if so, it seems to have been neglected.

In many of our larger eastern cities, mortuary records have been kept for many years past, and, from these, tables of mortality have been constructed. They merely, show, however, the number of deaths at different ages; but from them we do not learn the proportion of those that are born who die in infancy. We however know the positive infant mortality, and its proportion to that which occurs at more mature age.

The deaths in the city of New York, during fifty years, from 1804 to 1853 inclusive, was 363,242, and of these 176,103 occurred in children under five years. This is something less than one-half; but during the latter part of this period the proportion of infant mortality has been increasing, so that for many years past more than one-half have been under five years. Thus, in 1854, the total deaths under five years was 15,-673; and above five years, 12,865. In 1855, the total under five years was 14,063; and above that age, only 8,679. In 1856, under five years, 13,373; above, 8,285 During this latter year nearly five-eighths of the total number of deaths were under five years, and over one-half were under two years, and nearly three-eighths were under one year.

Though the proportion of infant mortality is greater in New York and the other large eastern cities, and is rapidly increasing there, while decreasing in most of the European cities, yet our own small cities and interior towns present pictures scarcely less appalling.

From a table of the deaths in Coldwater, during the year 1856 presented, at the last meeting of this body, by one of its most industrious members, Dr. J. H. BEECH, we learn that of the 70 cases, 38 were over five years, and 32 were under that age. From the records of the city sexton of Ann Arbor, the seat of our University, and one of the most healthy localities anywhere to be found, the deaths during the past year (1857) were 56, of which 22 were of persons over five years, and 34 under that age.

From the records of the city of Chicago for 1856, containing a population of 110,000, though somewhat imperfectly kept, and perhaps not altogether reliable, we gather that 1,950 deaths occurred, of which about 800 (something less than one-half) were under five years.

These facts are sufficient to show the enormous proportional extent of Disease and Death among these tender buds of humanity, and to commend the subject, on this account, to our consideration, as of special professional importance.

But can these results be avoided? Is it not in accordance with the plan of the Creator—within the designs of Providence—that a large portion of the human family should perish in their infancy—should thus be cut off before the full development of their being?

Although medical men are prepared to answer these questions in their own minds more promptly than others, yet perhaps, even among us, they require a respectful consideration. There can be no doubt of the fact that hygienic and medical management can alter these results; as well might we question whether the careless farmer, who so manages as to allow his young lambs to make their appearance in the dead of winter, and without proper protection leaves them to their almost inevitable fate, could have altered the result, by having them first open their little brutish eyes to the light of a vernal sun, and having for their first couch the warm earth, covered with Nature's carpet of green, instead of the frozen earth, covered with the winding sheet of snow.

About one hundred years ago, it was found, that in the imperfectly ventilated and improperly managed work-houses of London, of every *twenty-four* children born and retained in that establishment *twenty-three* died before attaining the age of one year! The subject was inquired into by parliament; an improved system of management was adopted, and the proportion of deaths was quickly reduced from 1,500 to 450 a year, thus saving annually, in these single establishment over 2,000 lives!

The more fully we realize, and the sooner all men and all women understand that disease and death are the result of fixed physical laws, and that most cases of sickness and *premature* death are the results of the palpable violation of these physical laws—the necessary penalties of physical wrong-doing—the better will it be for the human race; at least, if healthy existence is a blessing. That diseases are the results of the operation of natural laws, and not special Divine infliction, is no new doctrine among medical men; it is at least, as old as HIPPOCRATES, and has indeed ever had an existence among the eternal truths of nature.—There is a mistaken notion among many good people, which attributes all cases of sickness, injuries and death among their friends at least, to a

*Mysterious* Providence, though capable of being traced, with the utmost certainty, to violations of general and beneficent laws. I take, for illustration, a case of actual occurrence now present to my mind.

A mechanic was engaged in constructing a building. He was upon a scaffolding some distance above the ground, so built as to be capable of sustaining a weight, say, of three hundred and ninety-nine pounds. The weight of the man, and his building materials placed upon the scaffold, was, say, four hundred pounds. In accordance with the fixed law of attraction—of cohesion and gravitation—the scaffold gave way, and the man fell to the ground. The momentum acquired by the fall, his position, and the particular condition of the surface, resulted according to the same fixed laws, in a fracture of his leg. The poor man was conveyed to his home, where he was soon surrounded by his kind friends, and the venerable patriarch of the group spoke almost complainingly of a *Mysterious Providence* which had placed the good man, so much needed by his family, upon a bed of helpless suffering.

Now I do not accuse the sufferer in this case of having committed any moral offense—that is another and a distinct question; but, though committed ignorantly, I do accuse him of having violated the physical laws of GOD, established for the order and harmony of the Universe; and he was but suffering their just and necessary penalty in the injury inflicted. Instead of being a mysterious dispensation, it was the most natural, and, indeed, without a miracle, the only possible result of his own free act. But, suppose a miracle had been interposed, and, for his supposed puny accommodation, the general law of gravitation had been suspended;—planets and systems would have been thrown from their orbits, and the Universe would have fallen into dissolution. Or, if the law of gravitation had been suspended only as it related to his own body, he would have remained above the earth, and been thrown from its surface, while it whirled on its diurnal motion, and it would have passed entirely from him, leaving him in the broad waste of space as it flew on with lightning speed in the path of its annual orbit; and the poor man, if he could have collected his thoughts in time, would have cried out for the restoration of the law, though in its beneficent operation it had cost him the pain and danger of a broken leg.

I do not say the man was not laid upon his bed of suffering by the hand of Providence. He was bound there by that Being who has bound all nature fast, not in fate, but by law, and who, while not allowing a sparrow to fall to the ground without His notice, yet causes every sparrow to fall in obedience to the same law which holds the sun in the heavens.

I do not say that every casualty, sickness, or premature death is as easily traceable to an apparent cause as in the case I have instanced, to illustrate the principle; or that in every case evil results may be avoided as easily as they could have been in this. I do not say that in every case, sickness and premature death can, by any means, be avoided. From the unfavorable circumstances and errors of the ancestral race, there is often a feebleness of the vital force in individuals, which renders them incapable of enduring the ordinary vicissitudes of the most favorable hygienic circumstances, and with whom no care or skill can avert such consequences. Besides, there are contagious and other diseases which attack the most vigorous and best cared-for, which our present knowledge does not enable us to prevent. But I do say that all diseases are due to natural causes; that a very large proportion of them—that especially the enormous infant sickness and mortality which our statistics show to exist—are produced by causes which may be avoided, and which it is our special duty to search out, expose, and correct.

Let not what has been said respecting the fixed character of the physical laws, be misunderstood as tending to fatalism. The uniform and certain operation of these laws in no way interferes with the liberty of the human will, or the accountability of the free agent. The doctrine advanced, rather increases accountability, by extending it to acts under the physical laws.

I do not propose to pass beyond the limits belonging to a consideration of this subject from the stand-point of my own Profession. A special inquiry into the designs of the Creator in a theological and metaphysical sense, I shall leave, for the present at least, to those whose more special province it is to deal in such questions. It is sufficient for us to know that our business as physicians and our duty both as physicians and men, is to prevent and relieve human suffering, and save human life; and I have no reason for admitting the thought, that it was within the Divine plan that a large portion of the human family should be cut off in infancy, thus destroying so many specimens of handiwork, before their completion.

It would be a grave error, in my estimation, to suppose that the Creator designed that any of his laws should be violated; but it seems to have been in His design that, when any of those laws were violated, a penalty in kind should follow their transgression. The man or woman who, either knowingly or ignorantly, violates one of the natural or physical laws of his or her being; or the man woman, or child, in relation to whom, by whatever agency, a physical law is violated, in the fixed economy of the Universe must suffer a physical penalty—a penalty commensurate with the gravity and extent of the violation. The suffering of a physical penalty is irrespective and independent of any moral quality in the violation; but disobedience to physical laws becomes a moral offense when done consciously and willfully. As the guardians of helpless infancy, the moral responsibility is ours. We have no more reason for believing that the Ruler of the Universe desires the physical suffering and death of the physical transgressor, than that He desires the moral death of the moral transgressor. It is true that the result occurs in both instances from the operation of laws which He has established; but those laws were established for beneficent purposes—to develop animal and spiritual life and being, but not to destroy it.

It is sometimes fondly and poetically said, that certain little children, though ever so much the victims of mismanagement and consequent disease, are quite too pure for earth. In all given cases, it would perhaps be unkind, to rudely take from a bereaved parent the consolation which such a sentiment would afford; but whatever may be the condition of the innocent little spirit, the simple truth in relation to the material body is, that it is too corrupt for remaining on the earth. We certainly could not be justified in saying that, in any sense, the 2,150 infants under one year old which annually died in the work-houses of London, previous to the reform referred to, were more pure than the same number annually saved from death by that reform.

But it may be suggested (though it is impossible it should be by medical men), that, after all, it may be as well that children are thus removed from the evil to come. Are we thus to judge of Heaven's great gift of life? If such considerations are to abate efforts for preserving infantile life, let us say no more of the inhumanity of the sacrificing of children to heathen gods, or of the barbarity of casting them by hundreds into the waters of the Ganges. This speedy and almost painlesss method of destroying them is humane and merciful compared with the protracted suffering and lingering death which bad hygienic and medical management so often inflicts; far better would it be for the infant in New York to be cast into the Hudson to become food for the fishes, than to slowly die from want of pure air, of proper food, and of the light of heaven, whether it be in a bare and squalid cellar at the Five Points, or in a deeply-curtained and highly-perfumed chamber on Fifth Avenue; and then in the one case be cast, at the city expense, into a hole in the Potter's Field, and in the other to be solemnly deposited, surrounded by all the pomp and circumstance of magnificent woe, by the side of some tall family shaft in Greenwood. Though the grave of the one may be long strewn with flowers by the hand of affection, and its name be engraved in enduring marble, while the memory of the other is soon effaced even from its degraded mother's heart, yet the little bodies alike become the prey in the earth of the same noisome worms and decaying elements. The infant cut off from life before its conscious freedom is attained, is, of itself, but a blank in the world into which it has been introduced for other and higher purposes; and however diverse the circumstances of introduction and exit, to the same condition does each come at last. The handiwork of the Almighty has been destroyed—a beautiful casket, if it is regarded as nothing else, has been dashed to earth.

I have been led into these remarks, some of which may be regarded as digressive from the strict line of the subject, by the fact that it seems to be regarded by most persons as a matter of course, as proper as the order of nature and necessity, that a large portion of children should die; Many of the clergy preach thus, and all poets sing thus, and most people believe thus. I regard this sentiment as a grave error—a fatal heresy which has invaded the creed of humanity; for while such an opinion prevails, neither proper inquiries into the causes of infant mortality will be made, or efficient efforts will be put forth to prevent the effects of those causes. Every principle of political economy, of humanity, and of our enlightened and benevolent Profession, requires that the most careful inquiry and the most rigid efforts should be made.

But medical men should specially study the Diseases of Children because these diseases differ from those of adults. They can not be understood without this special study. They are different in their course and character, different in their appearances and treatment, and different in the methods by which they are distinguished. On these various points, however interesting, I do not propose specifically to dilate.

All those who distinguish carefully and watch closely the Diseases of Children, appreciate the force of the remarks just made. The expressions of the various suffering organs are widely different from those of the same organs in adults; and the helpless silence, the passionate agitation, and the fretful timidity of the child, combined very often with the exceedingly imperfect accounts of the attendants, sometimes hopelessly perplex even the acute physician, unless by study and care he has made himself perfectly familiar with the physiognomical appearances, pantomimic movements, and inarticulate sounds, as well as the physical signs expressive of different forms of disease, and also of the best methods of eliciting or observing them, and of overcoming all the difficulties to be encountered in the investigation. So great are these difficulties of diagnosis in many cases, that some parctitioners, after a few fruitless efforts, become satisfied with their ignorance, and declare it useless to attempt accurate diagnosis. It is unnessary to say that treatment without correct diagnosis must be quite in the dark, and, if at all active, mus- be iminently hazardous. In many cases the delicate system of the child illy bears perturbation, and the feeble spark of life many be easily extinghished. This delicacy of organization, and great susceptibility of medicinal impressions, while rendering the child more liable to be injured by rude or inappropriate treatment, renders him highly capable of responding to curative agencies, when nicely adjusted to the powers of endurance and the morditions.

It requires, then, more penetration to distinguish accurately the Diseases of Children than those of adults, and more skill to manage their cases in the most successful manner. If a professional subject increases in interest with its difficulty and its importance, then have the Diseases of Children the intensest professional interest. But do we all regard the subject in this light? Do we all take that interest in these diseases, and bestow that attention upon them they deserve? If the Profession every where did so, would not the complexion of our bills of mortality be altered? Do we not often, by our appearance of indifference in particular cases, intimate the neighborhood gossip, or some ready globulits, can manage these cases as well as ourselves?—and are we not thus instrumental in turning these children over to be drenched with the vile decoctions, or temporized with infinitesimals, when rational and skillful treatment is imperatively required?

If in this very imperfect presentation of this subject I have said any thing which shall arouse members of the Association to a higher sense of its importance, and call forth, to any extent, corresponding efforts, I shall have accomplished my most sanguine hopes, and the little labor I have bestowed upon this Report will not have been expended in vain.

[*The Peninsular and Independent Med. Journal.*]

